# Laparoscopic Loop Ileostomy for Severe Refractory Slow-Transit Constipation in a Patient With Charcot-Marie-Tooth Disease Type 2: A Case Report

**DOI:** 10.7759/cureus.116899

**Published:** 2026-09-25

**Authors:** Ishmam Murshed, Cassidy Campbell, Paul Heitmann, Jean Wong

**Affiliations:** 1 Department of Surgery, Royal Adelaide Hospital, Adelaide, AUS

**Keywords:** charcot-marie-tooth disease, colonic inertia, colorectal laparoscopic surgery, fecal diversion, gastrointestinal dysmotility, loop ileostomy, neuromuscular disease, slow-transit constipation, surgery

## Abstract

Severe constipation may cause profound disability despite daily bowel movements. Surgical decision-making is particularly challenging when gastrointestinal dysmotility coexists with neuromuscular disease and impaired functional independence.

A 60-year-old man with Charcot-Marie-Tooth (CMT) disease type 2 and wheelchair dependence spent four to five hours per day toileting despite osmotic laxatives and intermittent enemas. His distress and loss of independence had led him to seek access to voluntary assisted dying (VAD) services. Colonoscopy identified no obstructing colorectal lesion. Whole-gut transit testing showed preserved proximal transit, delayed colonic transit, and possible delayed rectal emptying. Formal anorectal physiology testing was not completed. Following shared decision-making with the patient, family, colorectal surgeons, and stomal therapy clinicians, he underwent laparoscopic loop ileostomy on April 2, 2026. Laparoscopy demonstrated minor omental adhesions, caecal adherence to the right abdominal wall, and dilated, flaccid bowel with reduced tone but some residual peristaltic activity. Early recovery included urinary retention. Ileostomy output decreased from 1.8 to 0.9 L/day after loperamide and psyllium were commenced. By postoperative day seven, he had changed his own appliance and was medically ready for discharge, pending stomal therapy clearance. At two months, he reported independent stoma management, bowel care taking minutes, and improved daily function and quality of life.

Slow-transit constipation and colonic inertia should not be treated as interchangeable diagnoses or as evidence of a specific neuromuscular lesion. Loop ileostomy may offer a potentially reversible diagnostic and therapeutic trial when the benefit of colectomy is uncertain, although longer-term follow-up is needed to assess its durable benefit.

## Introduction

Charcot-Marie-Tooth (CMT) disease comprises a genetically heterogeneous group of inherited motor and sensory neuropathies, with an estimated prevalence of approximately one in 2,500 people. Progressive distal weakness, sensory loss, muscle wasting, reduced reflexes, and foot deformity can impair mobility. CMT type 1 is predominantly demyelinating, whereas CMT type 2 is primarily axonal [1]. Gastrointestinal dysfunction is not a defining feature of CMT and should not automatically be attributed to the neuropathy. Reviews and case reports nevertheless describe digestive manifestations in selected patients, including constipation, colitis, esophageal dysmotility, and delayed gastric emptying. Autonomic or enteric involvement has been proposed, but the mechanisms and frequency of these manifestations remain uncertain [2,3,4].

In a questionnaire study comparing 58 patients with CMT and 54 healthy controls, Krhut et al. evaluated lower urinary tract, bowel, and sexual dysfunction collectively. The principal emphasis was on the increased burden of lower urinary tract symptoms; bowel symptoms were also more frequent in the CMT group. These findings support asking about bowel dysfunction alongside neurological and urinary symptoms, but do not establish objectively measured slow transit or show that CMT directly causes colonic inertia [5]. Slow-transit constipation is characterized by delayed colonic propulsion and is mechanistically heterogeneous; abnormalities involving enteric nerves, smooth muscle, and interstitial cells of Cajal have been described [6,7]. Delayed transit may coexist with a defecatory disorder or generalized gastrointestinal dysmotility. A transit abnormality therefore requires interpretation alongside evacuation symptoms and anorectal assessment, rather than being treated as proof of isolated colonic motor failure [8,9].

The American Society of Colon and Rectal Surgeons (ASCRS) conditionally recommends considering total abdominal colectomy with ileorectal anastomosis for selected patients with isolated refractory slow-transit constipation, and fecal diversion when other treatment options have failed [10]. The 2026 American Gastroenterological Association (AGA) expert review emphasizes adequate medical treatment, confirmation of slow transit, and assessment for defecatory and upper-gut disorders before surgery; temporary loop ileostomy may be considered when the likely benefit of colectomy remains uncertain [11]. We describe the assessment, operative findings, and early outcome of a laparoscopic loop ileostomy in a patient with CMT type 2 and disabling constipation.

## Case presentation

Clinical presentation

A 60-year-old man with CMT type 2, diagnosed at age 20, was referred for assessment of severe constipation of approximately eight years’ duration, which had been attributed clinically to an “atonic bowel.” He was wheelchair-dependent and had complex functional care needs. His Overall Neuropathy Limitations Scale (ONLS) score was 6, comprising an arm score of 2 and a leg score of 4. Although he opened his bowels daily, he passed only small amounts at a time and experienced persistent abdominal discomfort, bloating, and a sensation of incomplete evacuation. He reported spending up to four to five hours on the toilet each day.

His bowel regimen included polyethylene glycol (Osmolax), progressively increased over six years from two sachets once daily to two sachets three times daily, alongside Fleet enemas every two to three days for approximately six years, providing only partial relief. The burden of treatment and prolonged toileting had a profound effect on his independence, dignity, daily routine, and psychological well-being. The distress was such that he had accessed voluntary assisted dying (VAD) services, with this process being directed by a specialist VAD team comprising clinicians trained in VAD access and supply. This was addressed sensitively during shared decision-making. Preoperative psychiatric assessment included extensive review by two psychiatrists specialising in chronic disorders, informed by longitudinal psychology and neurology notes. The goal of operative management was not to cure the underlying bowel dysfunction, but to achieve meaningful symptom relief, reduce the toileting burden, and improve day-to-day quality of life.

He also reported intermittent rectal bleeding. His relevant surgical history included emergency laparoscopic omental patch repair of a perforated duodenal ulcer and previous open inguinal hernia repair. His medications included an osmotic laxative, esomeprazole, paracetamol, and nitrofurantoin for urinary tract infection prophylaxis in the setting of neurogenic bladder. He used prescribed medicinal cannabis and did not consume alcohol. There was no family history of colorectal cancer.

At surgical review, further nonoperative optimisation, additional physiological investigation, and potential stoma formation were discussed. The clinical presentation and whole-gut transit findings were considered more consistent with severe colonic slow-transit dysmotility in the context of Charcot-Marie-Tooth disease than with an isolated evacuatory disorder. The advantages and disadvantages of ileostomy and colostomy were explained. Given the concern regarding impaired propulsion through the colon, ileostomy was considered more physiologically appropriate than colostomy. The patient believed that he could manage a stoma and requested formal stomal therapy education before making a final decision.

Formal anorectal physiology testing, including anorectal manometry and balloon-expulsion testing, was considered but not performed. These investigations were judged unlikely to alter the planned loop ileostomy, which aimed to reduce the bowel-care burden and improve quality of life rather than to proceed with definitive colonic resection. Arranging these tests would also have delayed surgery and prolonged the patient’s substantial symptoms and daily toileting burden. Nevertheless, they could have identified coexisting defecatory dysfunction or altered rectal sensation. Their absence left rectal function incompletely characterised and remains a limitation of the diagnostic assessment.

Diagnostic assessment

Bowel preparation was adequate, and colonoscopy was completed to the caecum, with successful intubation of the terminal ileum. Findings included haemorrhoids, a normal terminal ileum, mild tortuosity of the transverse colon, and small-mouthed diverticula in the sigmoid and descending colon. Polyps at the hepatic flexure and in the sigmoid colon were biopsied and confirmed as benign hyperplastic polyps. No obstructing colorectal lesion or inflammatory process was reported. Haemorrhoids were considered the most likely explanation for the intermittent rectal bleeding. Figure 1 presents representative images from the colonoscopy.

**Figure 1:**
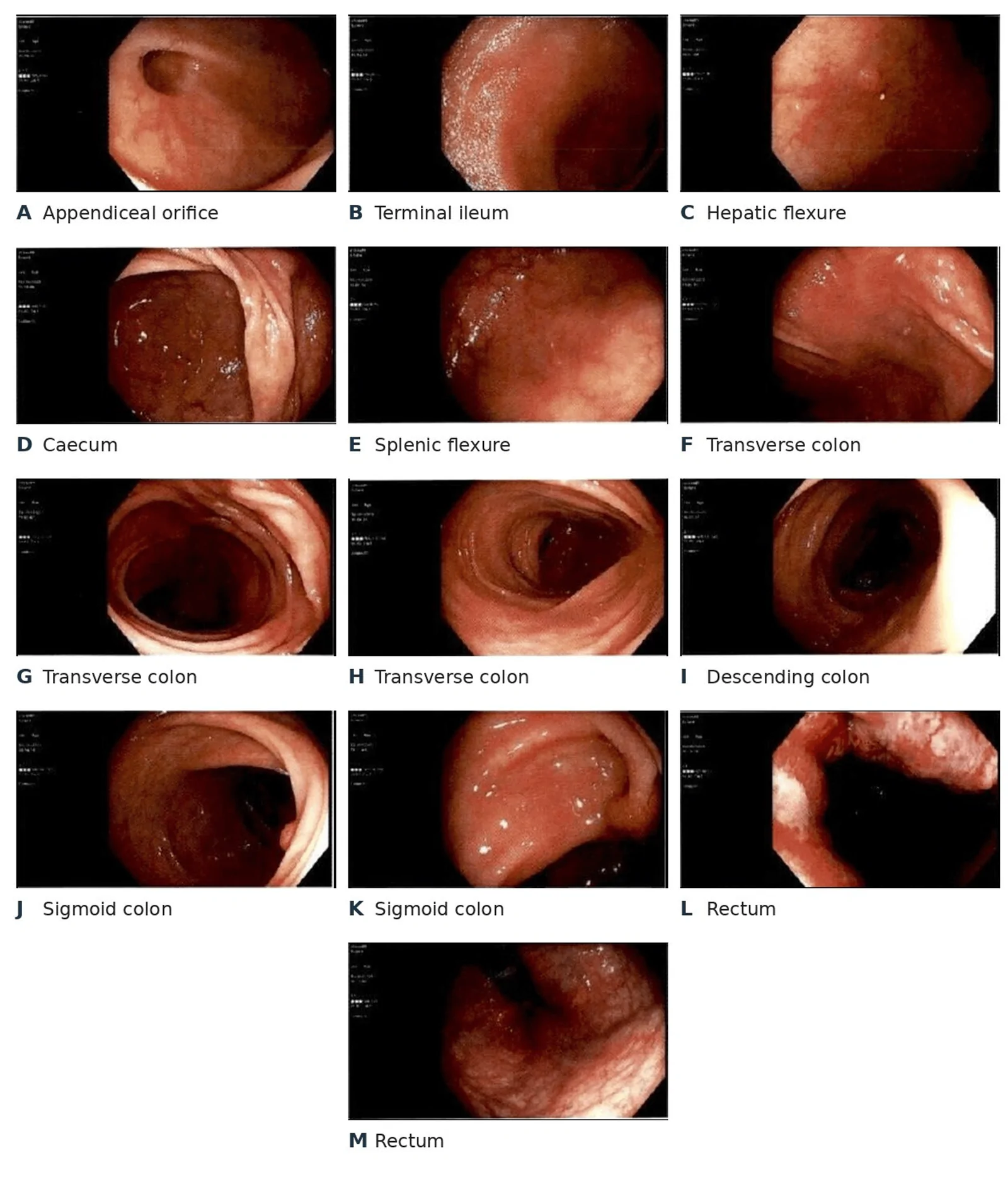
Representative colonoscopy images (A) Appendiceal orifice, with no focal abnormality reported; (B) normal terminal ileum; (C) hepatic flexure, where a 6 mm sessile polyp was reported and removed by cold-snare resection; (D) caecum, with no focal abnormality reported; (E) splenic flexure, with no focal abnormality reported (location supplied by the author); (F-H) transverse colon, reported as mildly tortuous; (I) descending colon, with small-mouthed diverticula reported; (J-K) sigmoid colon, with diverticula and two polyps reported, a sessile polyp measuring < 5 mm and an 8 mm semi-pedunculated polyp; and (L-M) rectum, with mild internal haemorrhoids reported. The remaining colonic mucosa was reported as normal. Images were collated from the official colonoscopy report completed before operative management

Whole-gut transit testing was undertaken to determine whether dysmotility was isolated to the colon or involved more proximal gastrointestinal segments. Oesophageal clearance was normal across three swallows. Gastric emptying was normal for both solid and liquid components. Tracer first entered the caecum within two hours, and approximately 44% of indium-111 activity had reached the terminal ileum and/or colon at six hours, meeting the department’s normal threshold of > 40%, in accordance with the Society of Nuclear Medicine and Molecular Imaging (SNMMI)/European Association of Nuclear Medicine (EANM) guidelines. These findings reduced concern that an ileostomy would be undermined by substantial gastric or small-bowel dysmotility.

Colonic transit was markedly delayed. At six hours, activity was visible in the ascending and transverse colon. At 48 hours, most activity remained centered around the splenic flexure, with only a small amount reaching the rectum. By 72 hours, most activity had cleared the colon and was located in the rectum. At 96 hours, most activity had been excreted, with a small amount of residual tracer remaining in the rectum. Quantitative analysis demonstrated delayed colonic transit, with a 50% emptying time of approximately 85 hours. The report concluded that small-bowel transit was within normal limits and that the colonic pattern was consistent with slow-transit constipation, with possible delayed rectal emptying. Table 1 provides a summary of the whole-gut transit findings.

**Table 1:** Summary of whole-gut transit findings

Segment/time point	Finding	Clinical interpretation
Esophagus	Normal clearance across three swallows	No objective evidence of esophageal transit delay
Stomach	Normal solid and liquid gastric emptying	No objective evidence of gastroparesis
Small bowel	Tracer entered the cecum within two hours	Small-bowel transit preserved
Colon: six hours	Activity in the ascending and transverse colon	Early progression into the colon
Colon: 48 hours	Most activity centered around the splenic flexure; small amount in the rectum	Markedly delayed colonic progression
Colon: 72 hours	Most activity had cleared the colon and was within the rectum	Delayed colonic transit with distal accumulation
Colon: 96 hours	Most activity excreted; small residual amount in the rectum	Possible delayed rectal emptying
Quantitative analysis	Colonic 50% emptying time approximately 85 hours	Consistent with slow-transit constipation

Differential diagnosis and procedural selection

The principal diagnostic challenge was distinguishing isolated colonic slow-transit constipation from a distal defecatory disorder, combined colonic and rectal dysfunction, mechanical colorectal pathology, or generalized gastrointestinal dysmotility. Colonoscopy did not identify an obstructing colorectal lesion. Preserved esophageal clearance, gastric emptying, and small-bowel transit supported consideration of ileal diversion, while markedly delayed colonic transit and the documented intraoperative finding of an enlarged, atonic bowel were consistent with predominantly colonic dysfunction.

A limitation of the preoperative assessment was the absence of anorectal manometry, balloon expulsion testing, and defecography. Residual rectal tracer suggested possible delayed emptying, but anorectal function remained incompletely characterized. Consequently, a coexisting defecatory disorder could not be confirmed or excluded, limiting certainty about the relative contribution of colonic and rectal dysfunction and the optimal surgical approach.

The decision to proceed with staged diversion took into account objectively demonstrated, markedly delayed colonic transit, alongside the patient’s wheelchair dependence, prolonged toileting burden, psychological distress, previous abdominal surgery, complex care needs, and preference for symptom relief. These considerations informed the individualized decision but did not resolve the limitations of the physiological assessment.

Shared decision-making and treatment

The case was reviewed with the patient and his family, colorectal surgeons, and stomal therapy clinicians. The rationale for surgery was to divert a poorly functioning colon rather than perform definitive resection. The patient was counseled that diversion could not guarantee complete resolution of abdominal discomfort, bloating, or all symptoms potentially arising from a broader neuromuscular disorder. He remained highly motivated to proceed because of the severity of his symptoms and their effect on his independence and quality of life.

Total abdominal colectomy with ileorectal anastomosis was discussed but was not selected initially. The team was concerned that possible delayed rectal emptying could leave the patient with ongoing difficult evacuation after colectomy. His previous laparotomy, functional care needs, and the potential consequences of an anastomotic complication were also considered. These concerns favored an initial diversion that preserved the colon. The team considered colostomy less suitable because stool passage would continue to rely on the colon, the principal segment with delayed transit in this patient. Cecostomy or antegrade irrigation was also considered less compatible with his upper- and lower-limb impairment and his goal of reducing the daily burden of bowel care.

Loop ileostomy was selected to bypass the colon, avoid an intestinal anastomosis, and preserve the possibility of later reassessment of surgical options. The patient underwent preoperative stomal therapy assessment, education, and site marking. Counseling covered the potential need for conversion to open surgery, bleeding, infection, bowel injury, parastomal hernia, prolapse, retraction, stenosis, peristomal skin complications, high output, dehydration, electrolyte disturbance, and renal impairment. Table 2 details the operative strategies considered and the rationale for selection.

**Table 2:** Operative strategies considered and rationale for selection

Option	Potential advantage	Reason not selected as the initial strategy
Total abdominal colectomy with ileorectal anastomosis	Definitive removal of a poorly propulsive colon in carefully selected isolated slow-transit constipation	Irreversible; dependent on satisfactory rectal evacuation; possible delayed rectal emptying; anastomotic and functional risks
Colostomy	Avoids small-bowel stoma and may produce more formed output	Continues to depend on propulsion through a colon with markedly delayed transit
Cecostomy or antegrade irrigation	Colon-preserving option that may facilitate scheduled evacuation	Ongoing irrigation and care requirements were less compatible with the patient’s functional limitations and desire for reliable diversion
Loop ileostomy	Bypasses the colon, avoids an anastomosis, preserves future options, and provides a staged assessment of response	Risk of high output, dehydration, electrolyte disturbance, renal impairment, and other stoma complications

Operative technique

The patient underwent laparoscopic loop ileostomy formation under general anesthesia, with regional and local anesthesia also recorded. Given the previous midline laparotomy, access was obtained using a modified Hasson cutdown technique through an 11-mm port in the left iliac fossa. Pneumoperitoneum was established, and two 5-mm ports were inserted under direct vision in the suprapubic and periumbilical positions.

Laparoscopy demonstrated minor omental adhesions to the previous midline laparotomy scar and adherence of the cecum to the right abdominal wall. The bowel loops were diffusely dilated, with a flaccid, boggy appearance and reduced tone on handling, although some peristaltic activity was observed during inspection. Omental adhesiolysis was performed using diathermy. An ileal loop was identified laparoscopically, and its orientation was confirmed before exteriorization through a trephine at the premarked right-sided stoma site.

The left iliac fossa fascial defect was closed with 0 Maxon. Local anesthetic (20 mL) was infiltrated into the port sites, and the skin was closed with 3-0 Monocryl. The loop ileostomy was matured with 3-0 undyed Vicryl. Operative time was 90 minutes, and estimated blood loss was less than 50 mL. No intraoperative complications were recorded.

Postoperative course and follow-up

On postoperative day one, the stoma was well perfused, and the patient tolerated oral intake without nausea or vomiting. Urinary retention occurred after a trial of void. By postoperative day three, he felt well, and the stoma appeared healthy. By postoperative day four, he was voiding well without nausea or vomiting.

On postoperative day five, ileostomy output over the preceding day was 1.8 L, prompting commencement of loperamide 2 mg three times daily and psyllium (Metamucil) 3.5 g. Psyllium was added with the aim of reducing output volume and limiting appliance leakage by improving effluent consistency. Renal function and serum electrolytes were monitored with daily morning blood tests; creatinine and potassium remained within the laboratory reference ranges. Output decreased to 0.9 L by review on postoperative day six and 625 mL by day seven. He had independently changed his appliance on day six and was medically ready for discharge by day seven, pending stomal therapy clearance, with a plan to omit the midday loperamide dose. Table 3 summarizes the clinical course.

**Table 3:** Postoperative timeline relative to ileostomy formation

Time point	Event or documented outcome
D0	Laparoscopic loop ileostomy. Minor omental adhesions to the previous midline scar and caecal adherence to the right abdominal wall. Diffusely dilated, flaccid bowel with reduced tone and some peristaltic activity. Omental adhesiolysis and maturation of a right-sided loop ileostomy
D1	Well-perfused stoma; oral intake tolerated. Urinary retention after a trial of void
D3-D4	Felt well with a healthy stoma by D3. Voiding well without nausea or vomiting by D4
D5	Previous day’s output 1.8 L; loperamide and psyllium commenced. Stoma education and discharge planning continued
D6	Previous day’s output 0.9 L; continued loperamide and stomal therapy. Participating in appliance changes
D7	Previous day’s output 625 mL; had changed his own appliance. Medically ready for discharge home, pending stomal therapy clearance, with a plan to omit the midday loperamide dose
Two months after surgery	Reported independent appliance management, bowel care taking minutes, and improved daily function and quality of life

At the two-month follow-up, the patient reported substantially easier bowel management. He managed the stoma independently, with appliance emptying taking only minutes compared with the prolonged toileting required before surgery. He described improvements in daily function, independence, dignity, and quality of life.

Ongoing follow-up was planned to monitor stoma output, oral intake, hydration, renal function, electrolytes, appliance management, peristomal skin health, complications, readmissions, and quality of life. Longer-term review was planned to assess the durability of these improvements and the patient’s preference for continued diversion or consideration of further surgery.

Patient perspective

“Before the operation, daily life was a genuine struggle. From the time I woke up until I went to bed, my bowels and stomach caused problems. Every morning I had to force down large amounts of laxative powder with water just to get some relief. There was constant pain, even after I managed to open my bowels. It was frustrating and exhausting, and I felt that my life was in a stranglehold. It affected my independence, dignity, and mental health. Things had become so difficult that I had accessed voluntary assisted dying services because I did not feel that I could continue living with that level of distress. Since the ileostomy, things have improved substantially. I can empty the bag when I need to, which is quick and much easier than what I dealt with before. I can manage the appliance myself, and I no longer have to sit and strain on the toilet. Overall, I am happy with the outcome. The stoma has made daily life much more manageable and has relieved symptoms that had become overwhelming. Because things have improved so much, I am no longer considering voluntary assisted dying."

## Discussion

This patient reported severe evacuation difficulty and loss of independence despite daily bowel movements. The clinical target was the time and distress associated with bowel care. Delayed colonic transit was demonstrated, but a direct causal relationship between CMT type 2 and the transit abnormality cannot be established from this case. Reports of bowel symptoms in CMT provide a relevant clinical context, while proposed autonomic or enteric mechanisms remain incompletely characterized [2,5].

Slow-transit constipation and “colonic inertia” should not be treated as interchangeable terms or as proof of a specific neuromuscular lesion. Enteric neural, smooth-muscle, and interstitial-cell abnormalities have been reported in slow-transit constipation, but their presence cannot be inferred in an individual from tracer retention alone [6,7]. In this patient, normal proximal transit and delayed colonic transit supported predominantly colonic dysfunction. Although the bowel appeared dilated and flaccid at laparoscopy, residual peristaltic activity indicated some preserved motor activity despite reduced tone. This highlights that markedly delayed transit can coexist with residual movement; however, visual inspection alone cannot establish effective propulsion or confirm or exclude colonic inertia. Residual rectal tracer also left open the possibility of a coexisting evacuation disorder.

Preserved upper-gut transit was relevant to the rationale for ileal diversion because severe small-intestinal dysmotility can create continuing nutritional and fluid-management difficulties despite operations directed at the colon [12]. A normal transit study does not exclude every form of dysmotility, and the patient’s early response cannot establish that proximal gastrointestinal function will remain unaffected. Colectomy with ileorectal anastomosis is an option for selected patients with isolated, medically refractory slow-transit constipation and satisfactory evacuation function. Published outcomes include persistent pain, diarrhea, incontinence, recurrent constipation, obstruction, and subsequent stoma formation, emphasizing the importance of careful patient selection and realistic expectations [8,9,10,13]. Formal anorectal testing was not completed here. This is a limitation of the workup, rather than evidence that such testing can routinely be omitted.

Current AGA advice supports excluding defecatory disorders and clinically significant proximal dysmotility before colectomy, and considering temporary loop ileostomy when the likely benefit of colectomy is uncertain [11]. In this case, surgery proceeded without completing the recommended anorectal evaluation. The resulting uncertainty about rectal function favored loop ileostomy as a potentially reversible diagnostic and therapeutic trial over irreversible colectomy. Diversion bypassed the colon and reduced reliance on rectal evacuation while avoiding an intestinal anastomosis and preserving anatomical options. However, improvement after diversion would not establish whether later colectomy or reversal would be successful; either would require further specialist assessment.

The choice not to pursue colostomy or antegrade irrigation reflected this patient’s transit pattern and functional goals. A colostomy would leave part of the delayed-transit colon in the fecal stream, while irrigation would retain an ongoing instillation and evacuation routine. These were case-specific concerns. The operation should not be presented as establishing that ileostomy is generally superior for all patients with neurological disability or constipation.

Direct evidence for surgical treatment of constipation in CMT remains limited in the literature reviewed here. A relevant neuromuscular comparison is the report by Glaser et al. of chronic intestinal pseudo-obstruction (CIPO) with predominant colonic involvement in a child with myotonic dystrophy, ultimately treated with total abdominal colectomy and end ileostomy, with no further admission for pseudo-obstruction reported over 17 months [14]. That case concerned a primary muscle disorder and recurrent pseudo-obstruction, whereas the present adult had an inherited peripheral neuropathy, delayed colonic transit, and preserved proximal transit. It supports individualized assessment but does not establish the best operation for CMT.

The broader neurogenic bowel literature offers a comparison for functional outcomes. Waddell et al. specifically reviewed colostomy after spinal cord injury, including 15 studies involving 488 patients. Nine of 11 studies assessing quality of life reported improvement, and all 13 assessing bowel-care time reported reductions; the evidence was predominantly retrospective [15]. Spinal cord injury differs from CMT in mechanism, and these colostomy outcomes do not establish the superiority of ileostomy. They nevertheless identify bowel-care time, independence, and satisfaction as useful outcomes alongside stool frequency, consistent with the improvements reported by this patient.

In a retrospective series of 87 patients with refractory constipation that did not fit classical physiological categories, Hung et al. reported that 54 (62%) met a composite outcome of subjective improvement, discontinuation of constipation medication, and ileostomy output above 200 mL/day after diverting loop ileostomy [16]. These patients underwent detailed preoperative investigation and did not constitute a CMT cohort. The study supports discussing diversion in selected complex cases, but its design does not establish a reliable prediction rule for subsequent colectomy, nor does it compensate for incomplete anorectal assessment in the present patient.

Longer-term consequences require equal emphasis. In the series by Davis et al., 38 women received loop ileostomy for intractable constipation, with a median follow-up of 34 months. Among 37 respondents, 49% reported no regret and 27% minimal regret, while 55% of the cohort underwent further stoma-related operations [17]. These are regret and reoperation outcomes, not a proven cure rate. The selected, all-female cohort and different clinical context limit extrapolation to a man with CMT but reinforce the need to discuss subsequent procedures and continuing stoma care. The two-month follow-up in the present case is insufficient to assess comparable longer-term outcomes, including reoperation and delayed regret.

Assessment should integrate physiological findings with treatment history, psychological well-being, expectations, and practical self-care needs [11,18]. In a patient with CMT, coordinated input from colorectal surgery, gastroenterology or neurogastroenterology, neurology, stomal therapy, dietetics, and rehabilitation or occupational therapy can help address those domains according to need. Hand function, wheelchair posture, reach, transfers, abdominal contours, appliance handling, and available assistance are practical considerations for education and stoma siting [18,19]. Here, the patient and his family participated in planning with colorectal surgeons and stomal therapy clinicians. Wider specialty involvement should be guided by the needs identified during assessment.

Counseling should include the possibility of long-term or permanent diversion and further surgery, alongside appliance care, skin protection, body-image concerns, parastomal hernia, prolapse, retraction, stenosis, and obstruction [17,18]. The retained distal bowel may continue to require attention, including management of mucus discharge. A plan for stoma supplies, assistance if neurological function changes, and access to specialist review is particularly relevant when independence is a central treatment goal [18].

High-output ileostomy can lead to water, sodium, and electrolyte depletion and renal impairment; clinical impact depends on intake as well as output, with outputs above approximately 1.5-2.0 L/day commonly prompting assessment [20]. In this case, an output of 1.8 L in the preceding day was documented at postoperative day five review; loperamide and psyllium were commenced, and the next review recorded 0.9 L over the preceding day. Long-term follow-up should address output, oral intake, urine output, hydration, weight, renal function, electrolytes, and recognition of dehydration. Individualized advice on oral rehydration and medication requires clinical review rather than an instruction simply to drink more plain water [20].

Figure 2 brings together the assessment and management considerations discussed above, from structured bowel-program optimisation and physiological testing to individualized surgical selection and long-term follow-up. In this case, predominantly colonic transit delay, incompletely characterized anorectal function, and the patient’s functional goals informed the choice of staged ileal diversion. The framework illustrates how these factors may guide decision-making in neuromuscular disease; it is a proposed synthesis of the literature rather than a validated treatment algorithm.

**Figure 2:**
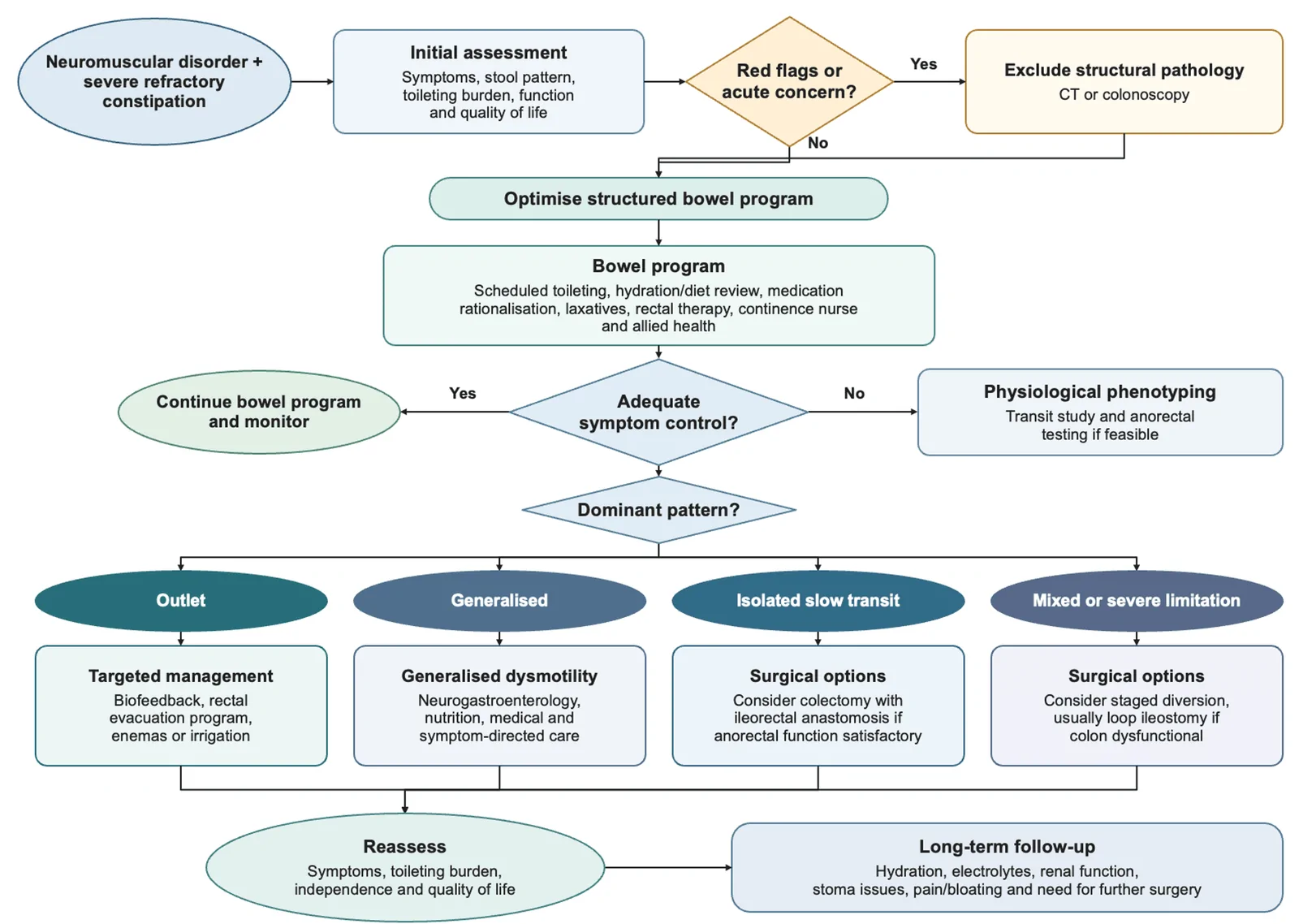
Proposed assessment and management framework for severe refractory constipation in neuromuscular disease The neuromuscular and Charcot–Marie–Tooth bowel-dysfunction context was informed by Krhut et al. [5]. The assessment sequence, structured bowel-program optimization, colonic transit testing, anorectal evaluation, and consideration of surgery were informed by Bharucha et al. [8], Alavi et al. [10], and Staller et al. [11]. The distinction between outlet dysfunction, generalized dysmotility, isolated slow transit, and mixed disease, together with caution regarding colectomy selection, was informed by Chaichanavichkij et al. [9]. Multidisciplinary assessment and management of generalized intestinal dysmotility were informed by Nightingale et al. [12]. The staged-diversion and loop-ileostomy pathway, including the need for long-term stoma counseling, was informed by Hung et al. [16] and Davis et al. [17] Image created by the authors

The patient’s account of prior access to VAD services conveys the severity of his individual distress. His reported change in outlook after diversion is relevant to his experience but does not demonstrate a causal effect of surgery on his decisions regarding VAD or replace assessment of psychological well-being. The report presents his perspective as an individual outcome.

The case report combines whole-gut transit findings, consideration of operative alternatives, documented laparoscopic findings, and a patient-reported early outcome. Laparoscopic observation of adhesions supports the operative description but does not establish colonic motor failure or its cause. Limitations include the absence of formal anorectal physiology testing, limited detail regarding prior medication trials, and the lack of prospectively collected validated measures of constipation severity or quality of life. Anorectal physiology testing could have helped characterise coexisting defecatory dysfunction [11].

Clinical response was assessed using documented symptoms, bowel function, and patient-reported improvement rather than validated instruments such as PAC-SYM, PAC-QOL, or the Cleveland Clinic Constipation Score [21-23]. Although renal function and electrolytes were monitored during admission, objective measurements of stabilized ileostomy output, electrolytes, and renal function were not reported at the two-month follow-up. This short follow-up also limits conclusions about optimal procedure selection and durable benefit. Longer-term assessment should incorporate validated symptom and quality-of-life measures alongside bowel-care time, functional independence, hydration and renal outcomes, readmissions, and stoma-related interventions.

## Conclusions

In this patient with CMT type 2, severe constipation caused prolonged and distressing evacuation despite daily bowel movements. Whole-gut testing demonstrated predominantly colonic transit delay, while possible rectal emptying dysfunction remained incompletely assessed. Laparoscopic loop ileostomy was selected through shared decision-making to reduce the bowel-care burden while preserving the colon. At two months, the patient reported easier bowel care and improved independence. This case report highlights that slow-transit constipation and “colonic inertia” are not interchangeable diagnoses, and neither alone establishes a specific neuromuscular lesion. Residual peristaltic activity observed during surgery illustrates that markedly delayed transit can coexist with preserved motor activity. When the benefit of colectomy remains uncertain, loop ileostomy may offer a potentially reversible diagnostic and therapeutic trial, although improvement after diversion does not establish the underlying cause or predict the likely outcome of colectomy or reversal. Incomplete anorectal assessment and short follow-up limit interpretation, and longer-term review is needed to assess durable benefit and stoma-related morbidity.

## References

[1] Burns J, Timmerman V, Laurá M (2026). Charcot-Marie-Tooth disease and related neuropathies. Nat Rev Dis Primers.

[2] Spagnoli C, Pisani F, Di Mario F, Leandro G, Gaiani F, De' Angelis GL, Fusco C (2018). Peripheral neuropathy and gastroenterologic disorders: an overview on an underrecognized association. Acta Biomed.

[3] Panunzio S, Couch L, Rahman A (2024). A case of Charcot-Marie-Tooth disease causing colitis and electrolyte imbalances. Cureus.

[4] Kloub M, Abusalim AI, Eldesouki M, Abushanab M, Okwesili B, Atiyat R, Bains Y (2025). Oesophageal dysmotility in a patient with Charcot-Marie-Tooth disease: report and literature review. Eur J Case Rep Intern Med.

[5] Krhut J, Mazanec R, Seeman P, Mann-Gow T, Zvara P (2014). Lower urinary tract functions in a series of Charcot-Marie-Tooth neuropathy patients. Acta Neurol Scand.

[6] Vlismas LJ, Wu W, Ho V (2024). Idiopathic slow transit constipation: pathophysiology, diagnosis, and management. Medicina (Kaunas).

[7] Tillou J, Poylin V (2017). Functional disorders: slow-transit constipation. Clin Colon Rectal Surg.

[8] Bharucha AE, Pemberton JH, Locke GR 3rd (2013). American Gastroenterological Association technical review on constipation. Gastroenterology.

[9] Chaichanavichkij P, Vollebregt PF, Tee SZ, Scott SM, Knowles CH (2021). Slow-transit constipation and criteria for colectomy: a cross-sectional study of 1568 patients. BJS Open.

[10] Alavi K, Thorsen AJ, Fang SH (2024). The American Society of Colon and Rectal Surgeons clinical practice guidelines for the evaluation and management of chronic constipation. Dis Colon Rectum.

[11] Staller K, Neshatian L, Lembo A, Bharucha AE (2026). AGA clinical practice update on evaluation and management of refractory constipation: expert review. Clin Gastroenterol Hepatol.

[12] Nightingale JM, Paine P, McLaughlin J, Emmanuel A, Martin JE, Lal S (2020). The management of adult patients with severe chronic small intestinal dysmotility. Gut.

[13] McCoy JA, Beck DE (2012). Surgical management of colonic inertia. Clin Colon Rectal Surg.

[14] Glaser AM, Johnston JH, Gleason WA, Rhoads JM (2015). Myotonic dystrophy as a cause of colonic pseudoobstruction: not just another constipated child. Clin Case Rep.

[15] Waddell O, McCombie A, Frizelle F (2020). Colostomy and quality of life after spinal cord injury: systematic review. BJS Open.

[16] Hung LY, Hull TL, Cline MS, Valente MA, Steele SR, Gorgun E (2022). Diverting loop ileostomy in the management of medically refractory constipation cases not falling into classical categories. Dis Colon Rectum.

[17] Davis G, Chapple K, Brown SR (2021). Ileostomy for chronic constipation: a good idea or just asking for more trouble?. Colorectal Dis.

[18] Davis BR, Valente MA, Goldberg JE, Lightner AL, Feingold DL, Paquette IM (2022). The American Society of Colon and Rectal Surgeons clinical practice guidelines for ostomy surgery. Dis Colon Rectum.

[19] Salvadalena G, Hendren S, McKenna L (2015). WOCN Society and ASCRS position statement on preoperative stoma site marking for patients undergoing colostomy or ileostomy surgery. J Wound Ostomy Continence Nurs.

[20] Nightingale JM (2022). How to manage a high-output stoma. Frontline Gastroenterol.

[21] Frank L, Kleinman L, Farup C, Taylor L, Miner P Jr (1999). Psychometric validation of a constipation symptom assessment questionnaire. Scand J Gastroenterol.

[22] Marquis P, De La Loge C, Dubois D, McDermott A, Chassany O (2005). Development and validation of the Patient Assessment of Constipation Quality of Life questionnaire. Scand J Gastroenterol.

[23] Agachan F, Chen T, Pfeifer J, Reissman P, Wexner SD (1996). A constipation scoring system to simplify evaluation and management of constipated patients. Dis Colon Rectum.

